# Commentary on "The effect of sexually transmitted disease education via Instagram on the knowledge and attitudes of university students: a pilot study"

**DOI:** 10.1590/1806-9282.20251009

**Published:** 2026-01-09

**Authors:** Buğra Çetin

**Affiliations:** 1 Altınbaş University Medical Park Bahçelievler Hospital, Department of Urology – Istanbul, Turkey.

Dear Editor,

We read with great interest the article titled "*The effect of sexually transmitted disease education via Instagram on the knowledge and attitudes of university students: a pilot study*" by Gürkan et al. The authors deserve recognition for addressing a timely and critical issue in public health: sexual education among young adults. Their efforts to incorporate a contemporary and youth-oriented medium such as Instagram represent an important initiative in digital health promotion.

While the study offers promising insights regarding the potential of social media in health education, we wish to contribute several reflections that may be valuable for the interpretation and development of future research in this area.

First, the use of a knowledge scale with a Kuder-Richardson-20 (KR-20) reliability score of 0.60 raises concerns about internal consistency. According to psychometric standards, a reliability coefficient below 0.70 may limit the credibility of an instrument's results and its ability to measure the intended construct effectively^1^.

Second, while the study successfully assesses short-term attitudinal changes, it does not make use of validated instruments such as the Condom Use Self-Efficacy Scale or the Sexual Health Literacy Scale for Young Adults. Without psychometrically rigorous tools, it becomes difficult to compare outcomes with the prior literature or to ensure reproducibility^2^. Moreover, behavioral outcomes were not measured, which limits conclusions about real-world impact beyond knowledge and perception.

Third, the absence of discussion around user engagement metrics and Instagram's algorithmic behavior is a notable gap. Variables such as content reach, interaction frequency, and video view duration have been shown to influence learning and behavior change in similar digital interventions^3^. Prior research demonstrates that incorporating these metrics can improve the targeting and personalization of health messages^4^.

Additionally, focusing exclusively on Instagram may reduce the broader applicability of the findings. Platforms such as TikTok and YouTube are also commonly used by adolescents and young adults for health information. McCashin et al., for example, found that the TikTok content related to mental health generated high engagement, though it varied in accuracy^5^. Similarly, Lupton emphasized the importance of YouTube as a primary channel through which youth seek peer-generated health content^6^. Future studies might benefit from comparing platform effectiveness in reaching and influencing health behaviors.

Another area that merits consideration is digital health literacy. As outlined by Walton and Richardson, the ability to critically evaluate and act on online health information is essential for the success of any digital intervention^7^. Including a measure of digital health literacy in future studies would add depth and improve the contextual understanding of participants’ responsiveness.

In conclusion, we appreciate the authors’ efforts in exploring a modern, digital avenue for sexual health education. Their study adds value to a growing field and lays the groundwork for larger, more comprehensive investigations. By integrating validated tools, platform-specific metrics, and a broader scope of digital behavior, future research can offer more robust and generalizable insights.

## Data Availability

The datasets generated and/or analyzed during the current study are available from the corresponding author upon reasonable request.
